# A deep learning-based classification method for subclinical zonular laxity in AS-OCT images

**DOI:** 10.3389/fcell.2026.1848716

**Published:** 2026-06-18

**Authors:** Jialin Liu, Lujie Zhang, Shuaixin Lu, JingLi Liang, He Teng, Jing Sun, Kai Wen

**Affiliations:** 1 School of Computer Science and Engineering, Tianjin University of Technology, Tianjin, China; 2 Tianjin Key Laboratory of Retinal Functions and Diseases, Tianjin Branch of National Clinical Research Center for Ocular Disease, Eye Institute and School of Optometry, Tianjin Medical University Eye Hospital, Tianjin, China; 3 Tianjin Children’s Hospital, Tianjin, China

**Keywords:** anterior segment optical coherence tomography, cataract surgery, deep learning, human lens zonular, medical image classification, preoperative assessment

## Abstract

**Objective:**

In this study, we developed and validated a deep learning method for the detection and angular position identification of subclinical zonular laxity using anterior segment optical coherence tomography (AS-OCT).

**Methods:**

A total of 600 curated AS-OCT images from 536 patients (600 images) undergoing cataract surgery were evenly stratified into subclinical zonular laxity (n = 300 images from 297 patients) and normal control (n = 300 images from 239 patients) groups. Data were partitioned at the patient level to prevent data leakage, with 60% for training, 15% for validation, and 25% for testing. An additional five clinical cases were used for external validation. We implemented MDCL-Net, a novel classification framework integrating mask-aware feature enhancement, dynamic contextual feature aggregation.

**Results:**

The model achieved an accuracy of 79.72%, an area under the receiver operating characteristic curve (AUC) of 86.41%, and an F1-score of 78.93%. Ablation studies confirmed the contribution of each module, and in clinical validation, model-predicted zonular laxity ranges showed good agreement with intraoperative observations across five representative cases.

**Conclusion:**

This work presents the first deep learning method capable of both detecting and spatially localizing subclinical zonular abnormalities in AS-OCT images, demonstrating strong clinical applicability and potential as a reliable preoperative screening tool to enhance surgical planning and safety in cataract procedures.

## 1 Introduction

The human lens zonule forms a fine fibrous system connecting the equator of the lens to the ciliary processes. Its three-dimensional structure, called the “Zinn ring” (Bassnett, 2021; Pan et al., 2023), provides static support and dynamic adjustment (Knaus et al., 2021). Integrity of the human lens zonula is critical for ocular surgery. Zonular laxity or dehiscence may result in lens subluxation or dislocation, leading to visual impairment and increasing surgical risks (Kang et al., 2024). Additionally, undetected subtle relaxation of the lens zonules can substantially impair the precision in determining the effective lens position, ultimately culminating in unsuccessful outcomes in refractive cataract surgery. Thus, an accurate preoperative evaluation of the human lens zonule is crucial in cataract surgery.

Pronounced abnormalities of the lens zonule can be evaluated by observing the comparative anterior chamber depth, iris tremors, and lens tremors under slit-lamp examination as well as *via* diagnostic instruments (Moshirfar et al., 2017; Cheng et al., 2022). These include Ultrasound Biomicroscopy (UBM); next-generation AS-OCT, exemplified by CASIA2; and the Scheimpflug camera-based three-dimensional anterior segment analyzer (Pentacam). However, the current modalities are hampered by substantial limitations in detecting subclinical zonular abnormalities. Although UBM can better visualize structures around the ciliary body from certain angles, its invasive nature requiring probe contact and lower resolution compared to AS-OCT limit its routine preoperative application. (Dagi and Walton, 2006). In contrast, AS-OCT provides non-contact, high-resolution imaging of anterior segment structures, making it more suitable for standard clinical workflow (Ang et al., 2018; Garcia Marin et al., 2022). In its standard imaging protocol, CASIA2 (Tomey Corporation, Nagoya, Japan) captures 16 radial cross-sectional scans per eye to achieve 360° circumferential coverage of the anterior segment. This acquisition scheme provides comprehensive circumferential assessment while maintaining clinical efficiency. However, subtle zonular abnormalities may still be missed if they fall between the scanned meridians. (Chen et al., 2022). Pentacam measurements remain vulnerable to artifacts owing to inherent lens elasticity and ciliary body morphological differences.

Given these limitations, an urgent need exists for more precise, objective, and automated diagnostic approaches. Deep-learning methods have shown remarkable capabilities in medical imaging tasks, including feature extraction, structural identification, and quantitative analysis, often surpassing manual methods in terms of sensitivity and reproducibility (Xu and Yang, 2023; Wang et al., 2025; Yang et al., 2025). These advantages make deep learning a promising tool for improving the evaluation of the zonular status. Based on this potential, this study proposes an accurate method (MDCL-Net) for the preoperative identification of subclinical zonular laxity.

## 2 Materials and methods

This study used clinical case imaging resources from the Tianjin Medical University Eye Hospital and employed standardized preoperative AS-OCT image data acquired using a CASIA2 device (Tomey Corporation, Nagoya, Japan). The CASIA2 device was used in its standard 16-scan radial acquisition mode, capturing images at 11.25° intervals around the corneal vertex to achieve 360° circumferential coverage.

A total of 536 patients (595eyes) were enrolled in this study between January 2023 and December 2024. Table 1 presents the detailed demographic characteristics of the study population.

**TABLE 1:** Demographic characteristics of the study population.

Characteristic	Zonular laxity	Normal control
Number of patients	297	239
Number of eyes	295	300
Age (years)	68.4 ± 8.7	65.2 ± 7.3
Sex (male, %)	47.1	52.3
Laterality of the eyes (right, %)	49.2	50

### 2.1 Diagnostic criteria and ground truth establishment

The subjects were stratified into subclinical zonular abnormalities and normal zonule groups based on the intraoperative assessment of zonular integrity, with documentation of the extent of the observed zonular pathology. The intraoperative assessment was performed by two experienced ophthalmic surgeons (JL.L. and H.T., each with more than 15 years of experience in cataract surgery). In cases of disagreement, a third senior surgeon (J.S., with 20+ years of experience) provided the final adjudication.

Subclinical zonular abnormalities—defined as zonular dialysis or laxity—were diagnosed according to four intraoperative signs: (1) asymmetric distribution of the anterior capsular folds during continuous curvilinear capsulorhexis, characterized by absent folds in the affected quadrants; (2) decreased anterior capsular tension with exaggerated equatorial mobility toward manipulation during CCC in the compromised areas; (3) decentration of the intraocular lens (IOL) accompanied by rotational difficulty during implantation; and (4) indentation of the capsular bag during cortical aspiration in the zones of zonular weakness. Meeting any one of these four criteria was considered sufficient for a diagnosis of zonular laxity, consistent with established clinical guidelines for intraoperative zonular assessment (Fairaq et al., 2025). Examination of the lens equator in the affected regions confirmed zonular dialysis when no zonular attachment was visible. Contrastingly, preserved zonules with excessive mobility indicated zonular laxity. The exclusion criteria were concurrent ocular pathologies (e.g., glaucoma), previous ocular surgery (including vitrectomy or refractive procedures), and preoperative slit-lamp evidence of partial lens subluxation manifested as iridodonesis, visible lens equator without dilation, or significant asymmetric anterior chamber depth.

This study was approved by the Human Research Ethics Committee of Tianjin Medical University Eye Hospital and adhered to the tenets of the Helsinki Declaration of 1975, as revised in 2000 (5). Informed consent was obtained from all patients included in the study.

### 2.2 Dataset partitioning strategy

To ensure rigorous evaluation and prevent data leakage, we implemented a patient-level partitioning strategy. All images from the same patient were assigned exclusively to one data split (training, validation, or testing) (Table 2). This approach ensures that the model’s performance reflects its ability to generalize to unseen patients rather than memorizing patient-specific imaging characteristics (Liu et al., 2023).

**TABLE 2:** Dataset distribution across training, validation, and test sets.

Split	Patients	Eyes	Images	Laxity	Normal
Training	299	343	348	174	174
Validation	82	86	86	43	43
Testing	155	166	166	83	83
Total	536	595	600	300	300

There was no patient or image overlap among the three splits. The training set was used for model parameter optimization, the validation set for hyperparameter tuning and early stopping, and the test set for final performance evaluation.

### 2.3 MDCL-net architecture

**FIGURE 1:**
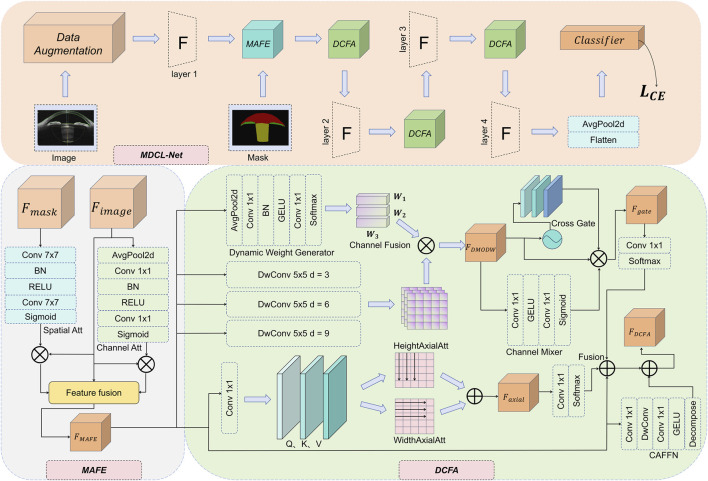
Overall architecture of MDCL-Net.

Based on our previous analysis, the classification task for subclinical zonular abnormalities faces two major challenges. First, due to occlusion by tissues such as the iris, the visibility of the zonular region is incomplete. Second, pathological alterations in regions such as the iris, anterior chamber angle, lens, and anterior chamber are subtle, making it challenging to effectively capture them using traditional methods. To address these challenges, this study proposes MDCL-Net framework for classifying latent zonular laxity (Figure 1).

The framework uses a progressive optimization strategy: First, the Mask-Aware Feature Enhancement (MAFE) module extracts mask information using a mask attention mechanism to locate key regions and suppress background interference. Next, the Dynamic Contextual Feature Aggregation (DCFA) module improves the details of each local region and integrates them with the global structural information to enhance the feature representation.

#### 2.3.1 MAFE module

In this subsection, we propose a method that enhances feature representations by integrating spatial and channel attention mechanisms *via* a dynamic feature fusion strategy that guides the model to locate critical regions while adaptively adjusting the channel-wise importance scores. The MAFE module comprises three key components.

Spatial Attention Branch: Leveraging mask guidance, this branch selectively enhances regions containing ocular anatomical structures (e.g., the anterior chamber, iris, angle, and lens) in the input feature maps *via* a spatial attention mechanism.

First, we concatenate the mask feature map $m\in R^{\text{Bx}1\text{xHxW}}$ with the sample feature map $x\in R^{BxCxHxW}$ along the channel dimension Equation 1:

$$x\in R^{\text{Bx}\text{Cx}\text{HxW}}$$ (1)

Subsequently, we apply two convolution layers to compute the spatial attention weights Equation 2:

$$\omega_{s}=\sigma \left({\text{Conv}}_{2}\left(\text{ReLU}\left(\text{BatchNorm}\left({\text{Conv}}_{1}\left(x_{s}\right)\right)\right)\right)\right)$$ (2)

Finally, spatial attention weights are applied to the input feature map to enhance it Equation 3:

$$x_{e,s}=x\cdot \omega_{s}+x$$ (3)

Channel Attention Mechanism: Based on channel-wise importance, we apply a channel attention mechanism to enhance informative channels of the feature map.

First, the input feature map x is compressed along the spatial dimension to 1 × 1 using adaptive average pooling Equation 4.

$$x_{c}=\text{AdaptiveAvgPool}2d\left(x\right)$$ (4)

Then, the channel attention weights are computed through two convolution operations Equation 5:

$$\omega_{c}=\sigma \left({\text{Conv}}_{2}\left(\text{ReLU}\left(\text{BatchNorm}\left({\text{Conv}}_{1}\left(x_{c}\right)\right)\right)\right)\right)$$ (5)

Finally, channel attention weights are applied to the input feature map to improve the feature map Equation 6:

$$x_{e,c}=x\cdot \omega_{c}$$ (6)

The original feature map x is subsequently combined with the enhanced channel attention features x_(e,c)_ along the channel dimension, followed by a 1 × 1 convolution to reduce channel dimensionality. The final output is obtained as the feature representation of the MAFE Equation 7:

$$f_{\text{MAFE}}={〖\text{Conv}〗}_{1\times 1}\;(\text{Concat}(x,\;x_{\left(e,s\right)},\;x_{(e,c)}))$$ (7)

#### 2.3.2 DCFA module

Human lens zonular laxity causes subtle alterations in the lens position and morphology, which may affect iris position, anterior chamber depth, and angle features (Kuchle et al., 2000; Kwon and Sung, 2017). Thus, we focused on quantifying alterations in the morphology and boundaries of these key regions, including:

1. Anterior chamber depth (distance from the corneal endothelium to the anterior surface of the lens).
2. Iris position (change in the relative position of the iris with respect to the lens and anterior chamber).
3. Anterior chamber angle (angular relationship between the cornea and iris).
4. Lens thickness (distance between the anterior and posterior surfaces of the lens).
5. Lens capsule curvature (quantified by surface curvature measurements).
6. The overall structural analysis (summarizes the alterations in the key structures while distinguishing unrelated regions).

We developed the DCFA module to address the challenge of these structures occupying only a small proportion of image pixels while ensuring the capability of the model to effectively capture these locally significant features.

The main objective of the DCFA module is to extract highly discriminative feature representations *via* multi-scale feature enhancement and geometry-continuity-aware modeling. This module incorporates multiple advanced techniques, including dynamic depthwise separable convolution, cross-gating mechanisms, and axial attention, to ensure the comprehensive capture of local details and global geometric structures in the AS-OCT images. The DCFA module mainly consists of two parallel branches.

##### 2.3.2.1 Local feature capture branch

The primary objective of this branch is to achieve cooperative multi-scale feature extraction *via* mechanisms such as multi-dilation rate depthwise separable convolution.

First, we use a multi-dilation rate depthwise separable convolution mechanism. A three-dimensional weight vector is generated *via* adaptive global average pooling, followed by a two-layer multilayer perceptron (MLP), which is subsequently normalized using Softmax and assigned to convolution branches with varying dilation rates. This approach effectively expands the receptive field while improving the modeling capability of key anatomical structures, such as angle configuration, anterior chamber depth, and lens morphology Equations 8, 9.

$$W_{M}=\text{Softmax}\left(\text{MLP}\left(\text{AdaptiveAvgPool}2d\left(f_{\text{MAFE}}\right)\right)\right)\in R^{B\times 3\times 1\times 1}$$ (8)

$$C_{\text{multi}}\left(f_{\text{MAFE}}\right)=\sum_{d=1}^{3}W_{M}\left(\text{DW}{\text{Conv}}_{d}\left(f_{\text{MAFE}}\right)\right)$$ (9)

Here, $W_{M}$ denotes a Softmax-normalized weight vector, $\text{DW}{\text{Conv}}_{d}$ represents a depthwise separable convolution with dilation rate $d\in \left\{3,6,9\right\}$ . In particular, depth-wise separable convolution focuses on capturing features at different scales, each focusing on various anatomical characteristics: geometric variations of the anterior chamber angle, distinctive changes in anterior chamber depth, curvature changes of the anterior and posterior lens surfaces, and geometric and positional features of the iris.

Subsequently, we use a cross-gating aggregation mechanism to integrate the local feature information extracted at varying dilation rates. This mechanism uses grouped shift-gating units to capture the spatial dependencies among the local features. Specifically, the grouped shift operation improves the spatial interactions between local features *via* feature shifting and gating strategies, enabling more precise extraction of the fine-grained details in critical regions Equations 10, 11.

$$S_{g}\left(C_{\text{multi}}^{\left(g\right)}\right)=\text{Roll}\left(C_{\text{multi}}^{\left(g\right)},\lfloor \frac{W}{G}\rfloor \cdot g\right),g=1,2,3,4$$ (10)

$$f_{\text{gate}}=ℶ\left(\text{MLP}\left(\sum_{g=1}^{4}{\text{Conv}}_{1\times 1}\left(S_{g}\left(C_{multi}^{g}\right)\right)\right)\right)⊙f$$ (11)

Here, $S\left(\cdot \right)$ is the grouped cyclic shift operation, where $G_{i}$ represents the number of groups (with g serving as the index). $ℶ\left(\cdot \right)$ refers to the channel-wise mixing weight modulation mechanism, and $f_{\text{gate}}$ is the output feature map of the local feature extraction branch.

##### 2.3.2.2 Geometric continuity capture branch

This branch focuses on modeling the global geometric structure in AS-OCT images by using an axial attention mechanism to capture the vertical and horizontal geometric relationships along the height and width dimensions, respectively. The input feature map is partitioned into multiple heads in this framework, with each head generating query(Q), key(K), and value(V) tensors for attention computation along distinct spatial orientations Equation 12.

$$Q,K,V=\text{Split}\left({\text{Conv}}_{1\times 1}\left(f_{\text{DAFE}}\right)\right)\in R$$ (12)

After the split operation, Q, K, and V are reshaped along the height and width axes to obtain the axis-specific tensors respectively. The vertical axis attention ${\text{Attn}}_{H}$ emphasizes structural features, such as anterior chamber depth and curvature of the anterior and posterior lens surfaces. Contrastingly, the horizontal axis attention ${\text{Attn}}_{W}$ prioritizes anatomical details, including angle configuration and iris morphology. This mechanism effectively extracts spatially discriminative relationships by explicitly modeling the geometric continuity of tissue structures, thereby enhancing the representation of the model Equation 13.

$${\text{Attn}}_{H}=\text{Softmax}\left(\frac{Q_{H}K_{H}^{T}}{\sqrt{d}}\right),{\text{Attn}}_{W}=\text{Softmax}\left(\frac{Q_{W}K_{W}^{T}}{\sqrt{d}}\right)$$ (13)

Where $d=\frac{C}{h}$ with h=4 is the scaling factor.

This formulation ensures that long-range dependencies are preserved while maintaining computational tractability, thereby enabling robust feature learning for classification. Here, the vertical attention weights ${\text{Attn}}_{H}$ mainly characterize the axial anatomical relationships from the corneal endothelium to the posterior lens capsule, whereas the horizontal attention weights ${\text{Attn}}_{W}$ encode the morphological features of the iris and the peripheral angle structures near the iris root. Cross-scale representation of the ocular anatomical structures depicted in AS-OCT images is achieved through the synergistic interaction of these dual-axial feature representation f_axial_ Equation 14.

$$f_{\text{axial}}=\text{Conv}\left({\text{Attn}}_{H}V_{H}+{\text{Attn}}_{W}V_{W}\right)$$ (14)

Subsequently, we propose a dynamic fusion gate mechanism to perform a weighted integration of the features derived from the local feature extraction and geometric continuity modeling branches. This mechanism enables adaptive weighting of local features and geometric structures based on their significance within the feature maps by computing fusion weights $\left(\alpha ,\beta \right)$ *via* softmax, thereby facilitating dynamic feature recalibration in the model Equations 15, 16.

$$\alpha ,\beta =\text{Softmax}\left(\text{Split}\left({\text{Conv}}_{1x1}\left(\left[f_{\text{gate}},f_{\text{axial}}\right]\right)\right)\right)$$ (15)

$$f_{\text{fusiongate}}=\alpha \cdot f_{\text{gate}}+\beta \cdot f_{\text{axial}}$$ (16)

Finally, the feature maps are further refined through a Channel Aggregation Feed-Forward Network to enhance the discriminative local features while performing feature decomposition $H\left(f_{\text{out}}\right)$ . This process effectively suppresses the interference from irrelevant background regions and reinforces the modeling of critical anatomical structures Equations 17, 18.

$$f_{\text{out}}=f_{\text{fusiongate}}+f$$ (17)

$$H\left(f_{\text{out}}\right)={\text{Conv}}_{1\times 1}\left(\text{GELU}\left({\text{Conv}}_{1\times 1}\left(f_{\text{out}}\right)\right)\right)$$ (18)

Here, $H\left(\cdot \right)$ represents the decomposition operator implemented *via* convolutional layers, and λ denotes the learnable scaling coefficient. The feature decomposition mechanism ensures the selective enhancement of critical information while suppressing irrelevant features. Finally, features from all branches are integrated *via* residual connections Equation 19.

$$f_{\text{DCFA}}=f_{\text{out}}+\lambda \cdot \left(f_{\text{out}}-H\left(f_{\text{out}}\right)\right)$$ (19)

The DCFA module achieves effective feature representation *via* three synergistic mechanisms:

1. Multi-scale feature coordination mechanism: Adaptive fusion of the local details and global geometric structures through dynamic depthwise separable convolution and cross-gating aggregation.
2. Axis attention mechanism: Precise modeling of spatial continuity in ocular anatomical structures *via* bidirectional vertical-horizontal geometric encoding.
3. Residual mechanism: Progressive feature refinement across deep networks ensured by a hierarchical information flow and gradient stabilization design.

For final classification, a fully connected layer is applied to the embedding representation. The model parameters are optimized by minimizing the cross-entropy loss using gradient-based optimization.

### 2.4 Evaluation metrics and statistical analysis

The data were partitioned at the patient level with the same ratio (60% training, 15% validation, 25% testing). Final results are reported as mean ± standard deviation under three random seeds.

We report the following metrics: Accuracy, Area Under the Receiver Operating Characteristic Curve (AUC), F1-Score, Sensitivity, and Specificity.

For comparison with conventional clinical diagnosis, we simulated the diagnostic performance of experienced ophthalmologists (with 10+ years of experience) based on preoperative slit-lamp examination alone, without intraoperative confirmation. This comparison demonstrates the added value of the proposed deep learning system over standard preoperative assessment.

## 3 Results

### 3.1 Parameter setting

The experimental framework was implemented using PyTorch, and model training was performed on an NVIDIA GeForce RTX2080Ti GPU. All input images were preprocessed to a standardized resolution of 406×255 pixels before processing using the ResNet-18 backbone architecture, which consisted of four residual blocks. Model optimization was conducted using SGD with a learning rate η of 0.01 and weight decay of $5\times 10^{-4}$ .

### 3.2 Experimental results and comparisons

We directly compared our model’s final classification accuracy, F1-score, and AUC values with those of other methods, including ST-ProtoNet (Wang et al., 2023), DiffMIC(Yang Y. et al., 2023), PPCA-Net (Zhang et al., 2024a), Mamba-Vision (Hatamizadeh and Kautz, 2025), and Transx-Net (Lou et al., 2025), to demonstrate the advantages of MDCL-Net. All the methods were assessed under identical experimental settings. Table 3 presents a comparative analysis of the classification performances of the proposed method and the aforementioned approaches for the AS-OCT dataset (mean ± SD across three random seeds).

**TABLE 3:** Performance comparison of different methods on the AS-OCT dataset.

Method	Year	AS-OCT		
		ACC (%)	AUC (%)	F1-score (%)
ST-ProtoNet	ICCV-2023	74.09 ± 1.80	82.31 ± 4.20	73.52 ± 0.43
DiffMIC	MICCAI-2023	73.40 ± 5.78	73.40 ± 5.78	72.91 ± 5.11
PPCA-Net	TNNLS-2024	74.69 ± 3.22	86.01 ± 2.14	73.46 ± 3.90
Mamba-Vision	CVPR-2025	73.69 ± 2.28	86.09 ± 1.53	76.82 ± 1.31
TransX-Net	TNNLS-2025	71.49 ± 1.52	78.18 ± 0.66	71.48 ± 3.11
MDCL-Net	Ours	79.72 ± 0.02	86.41 ± 1.65	78.93 ± 3.24

The MDCL-Net proposed in this study exhibited superior performance on the AS-OCT dataset for the classification of subclinical zonular laxity (Table 3). The MAFE mechanism strengthens the features of the target objects and accentuates their spatial localization, whereas the DCFA module captures subtle morphological differences in the target structures, enabling the network to extract more distinctive features.

### 3.3 Module ablation experiment

We conducted ablation experiments using the AS-OCT dataset to demonstrate the effectiveness of the MAFE, DCFA. These experiments were designed to analyze the impact of each module on the classification performance within the framework.

1. Baseline: Only the ResNet18 backbone and classifier were retained.
2. Baseline + MAFE: The MAFE module guides the model to localize the key target regions.
3. Baseline + DCFA: The DCFA module enhances the ability of the model to capture subtle variations.
4. Baseline + MAFE + DCFA: Combined effect of the MAFE and DCFA modules.

Our proposed modules achieved varying degrees of improvement in the classification accuracy on the AS-OCT dataset (Table 4).

**TABLE 4:** Ablation experiments conducted on the AS-OCT dataset.

#	MAFE	DCFA	ACC (%)	AUC (%)	F1-score (%)
(a)	×	×	72.09 ± 0.28	80.10 ± 0.27	71.96 ± 1.55
(b)	√	×	78.11 ± 2.22	84.84 ± 1.01	76.79 ± 2.88
(c)	×	√	73.49 ± 0.98	81.44 ± 3.51	75.99 ± 1.89
(d)	√	√	79.72 ± 0.15	86.41 ± 1.65	78.93 ± 3.24

### 3.4 Clinical validation and case analysis

**FIGURE 2:**
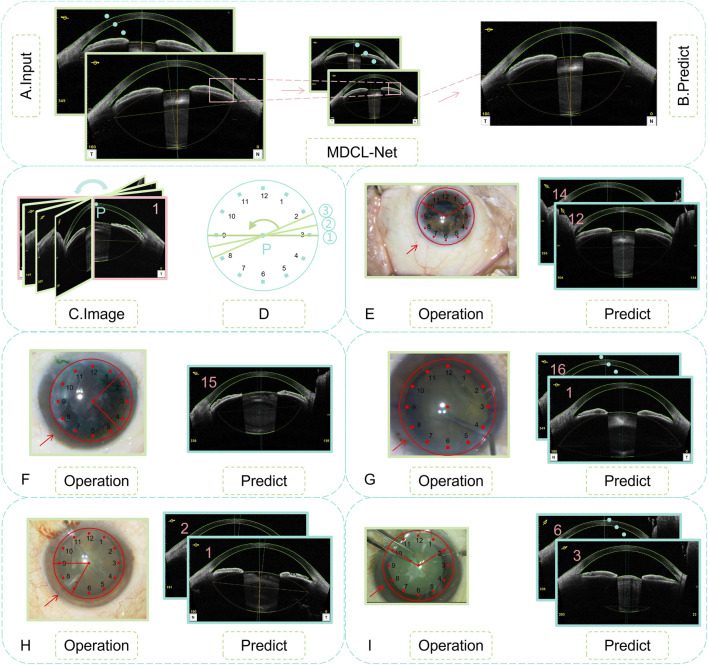
The application results of clinical cases.

To further validate the clinical applicability of MDCL-Net in diagnosing subclinical zonular laxity, we conducted a prospective case series analysis using AS-OCT images acquired under a standardized imaging protocol. In these cases, the surgeons did not detect any zonular laxity of the lens in the patients *via* slit-lamp examination before surgery. Due to the disorder of the numbers in Figure 2, we have adjusted the numbers in Figure 2 and moved the first sentence of the second paragraph here: The 16 AS-OCT images from each patient were input into the trained MDCL-Net model for inference (Figure 2A). The model classified each image and output the sequence numbers of those predicted as zonular laxity (Figure 2B). The image acquisition procedure was as follows (Figure 2C): the corneal vertex (point P) was set as the center, and the horizontal line connecting the nasal and temporal sides was defined as the reference baseline (Figure 2D). Starting from this baseline, AS-OCT images were captured counterclockwise around the corneal vertex (Z-axis) at 11.25° intervals, yielding 16 images per eye to achieve 360° circumferential coverage of the anterior segment. This acquisition scheme corresponds in a two-dimensional representation to images obtained from the three o’clock to nine o’clock positions. The 16 AS-OCT images from each patient were input into the trained MDCL-Net model for inference (Figure 2C). The model classified each image and output the sequence numbers of those predicted as zonular laxity (Figure 2D).

To translate the predictions into spatially meaningful clinical localization, we interpreted the output accordingly. For example, if the model identified images 1, 2, and 3 as laxity, this corresponded to an inferred zonular laxity within the angular ranges of 0°–33.75° and 180°–213.75°. The model-predicted laxity ranges were then compared with intraoperative observations during cataract surgery.

Based on available clinical data, we present five representative cases that met the same zonular assessment criteria used in model training. Zonular laxity was located between 10o and two o’clock in Patient A (Figure 2E), between 1:30o and five o’clock in Patient B (Figure 2F), across the entire circumference in Patient C (Figure 2G), between seveno and nine o’clock in Patient D (Figure 2H), and between 10o and two o’clock in Patient E (Figure 2I).

MDCL-Net analysis yielded the following predictions:

1. For Patient A, image #12, #14 was classified as abnormality, corresponding roughly to the 10o–11 o’clock position, which partially matched the surgical findings.
2. For Patient B, images #15 were predicted as laxity, generally aligning with the clinically observed local laxity range.
3. For Patient C, images #1 and #16 were classified as laxity, covering most of the circumference and matching the observed generalized laxity.
4. For Patient D, images #1 and #2 were identified as abnormality, corresponding to the clinically noted local laxity zone.
5. For Patient E, images #3, and #6 were predicted as abnormality, showing reasonable agreement with surgical observations.

Notably, in all five cases, the preoperative slit-lamp examination performed by experienced ophthalmologists did not reveal any signs of zonular laxity (e.g., iridodonesis, lens tremor, or asymmetric anterior chamber depth). However, our MDCL-Net model successfully classified each of these five cases as positive for subclinical zonular laxity, and the model-predicted angular ranges showed reasonable agreement with intraoperative observations. This direct comparison demonstrates that the proposed deep learning system can detect zonular laxity that is missed by conventional clinical examination, highlighting its potential as a sensitive preoperative screening tool.

### 3.5 Visualization results

**FIGURE 3:**
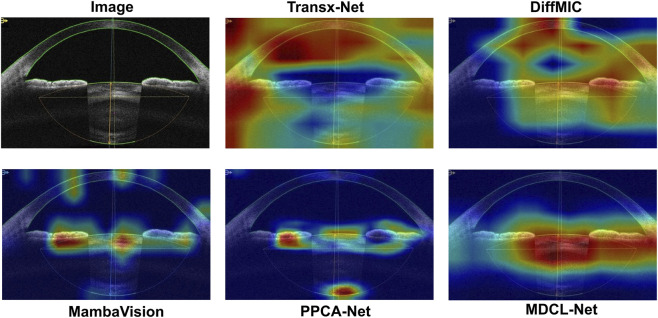
Comparative analysis on visualization results of methods in classification task of the human lens zonule.

We conducted a Class Activation Mapping (CAM) analysis of the feature maps generated by the last layer of the encoder to further validate the effectiveness of MDCL-Net. The CAM results provide an intuitive visualization of the feature regions captured by the network. In the CAM images, a deeper red indicates higher attention from the network to the corresponding region, whereas a deeper blue indicates lower attention. We randomly selected one image for the visualization analysis (Figure 3).

Based on the visual analysis results, our network effectively focused on and highlighted key target regions. Using the MAFE module, the network accurately captured the approximate locations of the anterior chamber, iris, and lens.

## 4 Discussion

Preoperative detection of subclinical zonular abnormalities enhances surgical safety, optimizes refractive outcomes, supports counseling, and ensures contingency planning (Sangal and Chen, 2014; Trikha et al., 2016). Specifically, undetected zonular laxity is associated with increased risks of intraoperative complications including capsular tear, vitreous loss, and dropped nucleus, which can lead to severe visual impairment. Identifying these abnormalities preoperatively allows surgeons to select appropriate surgical techniques (e.g., capsular tension rings, iris hooks) and IOL types, thereby reducing complication rates and improving visual outcomes.

In AS-OCT image analysis, substantial progress has been made in deep-learning architectures for ophthalmic disease evaluation, relevant studies are summarized in Table 5. Those disease-specific techniques (Hao H. et al., 2021; Hao J. et al., 2021; Zhang et al., 2022a; Zhang et al., 2022b; Zhang et al., 2022c; Karthik and Mahadevappa, 2023; Yang B. et al., 2023; Li et al., 2024; Zhang et al., 2024b) share the common characteristic of transforming clinical features from anatomical structures such as the anterior chamber, iris, and lens into differentiable learning objectives, effectively achieving an organic integration of medical priors and deep learning. However, current artificial intelligence applications for AS-OCT image analysis predominantly focus on directly visualized anatomical structures, whereas the detection of indirectly observable entities, such as the lens zonula, remains largely unexplored.

**TABLE 5:** Studies pertaining to AS-OCT image.

Author	Model	Contributions highlights
(Li et al., 2024)	$E^{2}GAN$	Introduced a contrast attention mechanism and gradient-guided filtering modules, the proposed method effectively achieves AS-OCT image denoising in an unpaired setting, significantly suppressing speckle noise whilst excellently preserving critical edge structures
(Karthik and Mahadevappa, 2023)	-	By replacing the standard residual connections in ResNet, this approach achieves significant enhancement of retinal layer features whilst effectively reinforcing minor gradients
(Zhang et al., 2022c)	CCA-Net	Proposed a clinical context-aware attention network, which extracts clinical features through hybrid pooling and constructs a clinical feature integration mechanism *via* channel-interactive fully connected layers
(Zhang et al., 2024b)	RCRNet	Proposed a region-context recalibration network, which extracts clinical features through region pooling and integrates them *via* a hierarchical recalibration mechanism
(Zhang et al., 2022b)	AFSNet	Developed an adaptive feature squeezing network to evaluate nuclear cataracts in AS-OCT images
(Yang et al., 2023a)	HA-Net	Proposed a hierarchical attention multi-task network based on U-Net for ciliary muscle segmentation in AS-OCT images
(Hao et al., 2021b)	HV-Net	Designed a hybrid variational perception network to assess anterior chamber angle (ACA) openness
(Hao et al., 2021a)	MSDN	Proposed a multi-sequence deep network that leverages spatiotemporal features from AS-OCT image sequences to classify ACA subtypes
(Zhang et al., 2022a)	RIR	Introduced a region-integrated recalibration attention (RIR)-based network for nuclear cataract grading, which dynamically adjusts the weights of the upper, lower, and central nuclear features by simulating uneven opacity distributions
(Yang et al., 2023b)	Hybrid CNN-Transformer	Proposed a hybrid CNN-Transformer model for automated ACA parameter measurement in AS-OCT images, featuring a dual-branch architecture that simultaneously captures local fine-grained features and global information from the iris root (IR) and scleral spur (SS)
(Fang et al., 2023)	-	Proposed a shape-prior-based deep learning framework for accurate segmentation of the lens nucleus and cortical layers in AS-OCT images, using a convolutional neural network to learn the level set function
(Xiao et al., 2024)	MSSANet	Introduced a multi-style spatial attention module, which extracts clinical-style features such as mean, maximum, and standard deviation *via* group-style pooling and improves cortical cataract classification *via* local transformation and feature recalibration mechanisms

The unobservability of the lens zonule presents novel challenges for deep learning methodologies in classification and measurement tasks, despite the fact that medical image analysis has substantially evolved from single-architecture processing to hybrid architectures, with a gradual shift from general-purpose designs to clinically optimized solutions (Pham et al., 2021; Huang et al., 2024; Shan et al., 2024; Zhang Y. et al., 2024; Lee et al., 2019). We propose an indirect evaluation method that analyzes the secondary manifestations of human lens zonular laxity, such as alterations in the lens position, anterior chamber depth, and lens morphology, to indirectly infer the status of the zonula. Previously, embedding domain knowledge derived from the physical properties of images into models has been shown to markedly enhance the fine-grained processing capabilities of medical image analysis tasks (Zhang et al., 2022c; Karthik and Mahadevappa, 2023; Li et al., 2024; Zhang et al., 2024a; Zhang et al., 2024b; Mao et al., 2025). However, our analysis revealed that traditional attention mechanisms struggle to simultaneously localize multiple anatomical structures. In addition, interference from unrelated regions can decrease the signal-to-noise ratio during feature extraction. To address these challenges, we developed the MAFE module. This module uses previous knowledge (anatomical masks) to guide the model to accurately locate key anatomical regions (such as the anterior chamber, angle, iris, and lens). This module combines spatial attention mechanisms to extract region-specific weighted maps and improve the localization accuracy of key regions. Subsequently, a channel-attention mechanism is utilized to filter the most informative feature channels.

These features are classified into low-resolution (such as anterior chamber depth, iris position, lens thickness, and overall morphological alterations in the anatomical structures) and high-resolution features (such as angle and lens capsule curvature changes). Based on these morphological and structural analyses, we propose the DCFA module. This module improves multi-scale feature representation by combining dynamic multi-stage deep convolutions, cross-gated aggregation, and axial attention mechanisms. This enables the model to precisely analyze local morphological alterations in each key region, aggregate local observations, and perform a comprehensive evaluation of human lens zonular laxity.

## 5 Conclusion

Herein, we proposed a novel classification method for the human lens zonula, MDCL-Net. This framework comprises three key components: a feature extractor, feature optimizer, and classifier. The feature extractor uses ResNet18 for feature extraction. Contrastingly, the feature optimizer improves the model’s feature representation capability by integrating the MAFE and DCFA modules, guiding the model to focus on target regions while improving its ability to capture detailed information. In most cases, our technique achieved state-of-the-art performance, and the experimental findings further validated its feasibility.

## 6 Limitations and future work

This study has several limitations that should be acknowledged. First, the dataset size (600 images from 536 patients), while carefully curated, is relatively modest for deep learning applications. Although we mitigated this through patient-level data partitioning, and extensive data augmentation, larger multi-center datasets would strengthen the generalizability of our findings. Second, the data were collected from a single center with a homogeneous ethnic population (East Asian), which may limit applicability to other ethnic groups with different anatomical characteristics. Third, all images were acquired using a single AS-OCT device model (CASIA2), and model performance on images from other devices remains to be validated. Fourth, while the model showed good performance in detecting the presence of zonular laxity, angular position predictions showed partial discrepancies due to the 11.25° scan interval limitation.

Future work will focus on: (1) expanding the dataset through multi-center collaborations to include diverse ethnic populations and imaging devices; (2) developing post-processing algorithms to interpolate between scanned meridians for improved angular precision; (3) conducting prospective clinical trials to validate the clinical utility of the system in routine preoperative workflows; and (4) integrating the model into real-time AS-OCT analysis software for point-of-care decision support.

## Data Availability

The original contributions presented in the study are included in the article/supplementary material, further inquiries can be directed to the corresponding authors.
